# Multiomics Analysis of Molecules Associated with Cancer in Mesenchymal-Stem-Cell-(MSC)-Derived Exosome-Treated Hepatocellular Carcinoma Cells

**DOI:** 10.3390/cimb46120793

**Published:** 2024-11-21

**Authors:** Wen-Yong Gao, Chantana Boonyarat, Nutjakorn Samar, Benjabhorn Sethabouppha, Pornthip Waiwut

**Affiliations:** 1 Faculty of Pharmaceutical Sciences, Ubon Ratchathani University, Ubon Ratchathani 34190, Thailand; gaowenyong@biocoso.com (W.-Y.G.); nutjakorn.sa.65@ubu.ac.th (N.S.); benjabhorn.s@ubu.ac.th (B.S.); 2 Faculty of Pharmaceutical Sciences, Khon Kaen University, Khon Kaen 40002, Thailand; chaboo@kku.ac.th

**Keywords:** UC-MSCs, HepG2 cells, RNA sequencing analysis, exosomal proteins, neutrophil extracellular trap formation

## Abstract

Hepatocellular carcinoma (HCC) is the most common form of liver cancer in humans, with an increasing incidence worldwide. The current study aimed to explore the molecular mechanisms that inhibit the proliferation of HepG2 cells, a hepatoblastoma-derived cell line. MSC-derived exosomes (UC-MSCs) were prepared with a median particle size (N50) of 135.8 nm. Concentrations of UC-MSCs ranging from 10 μg/mL to 1000 μg/mL were applied to HepG2 cell cultures and compared to untreated and anticancer drug-treated HepG2 cells. A combined approach was employed, integrating a proteomic analysis of UC-MSCs, metabolomic analysis of HepG2 cells, and transcriptomic profiling of HepG2 cells to decipher the inhibitory mechanisms of UC-MSC exosomes on HepG2 cell growth. Treatment with a high concentration of UC-MSCs led to a notable reduction in HepG2 cell viability, with survival decreasing by 65%. A proteomic analysis of UC-MSCs revealed enriched degranulation processes in Gene Ontology (GO) and Kyoto Encyclopedia of Genes and Genomes (KEGG) pathways, in addition to the known exosomal pathways. Transcriptomic profiling showed distinct changes in the expression of genes related to hepatocellular diseases in UC-MSC-treated HepG2 cells, contrasting with changes observed in HepG2 cells treated with the chemotherapeutic agent doxorubicin (DOX). Combined with a metabolomic analysis, the detailed GO and KEGG pathway analyses indicated that pathways associated with neutrophil extracellular trap formation played a critical role in mediating protein degradation and suppressing central carbon metabolism in cancer cells. Our results revealed that the UC-MSC treatment mimicked molecular mechanisms similar to those involved in neutrophil extracellular trap formation, exhibiting effects on HepG2 cell growth suppression that differed from those of chemical cancer drugs. Notably, the UC-MSC treatment demonstrated that protein degradation in HepG2 cells was regulated through canonical signaling pathways activated by bacterial peptides in neutrophils. This research has provided valuable insights into the potential of MSC-derived exosomes as a therapeutic approach for cancer treatment in the future.

## 1. Introduction

Over the course of several decades, the safety and feasibility of a number of exosome clinical applications were proven in clinical trials [1,2]. Various types of normal cells were able to produce exosomes, such as mesenchymal stem cells (MSCs), human umbilical vein endothelial cells, and immune cells. Cancer cells can secrete large amounts of exosomes, which play a crucial role in cancer proliferation, migration, and invasion. MSCS are pluripotent stem cells that are able to adapt to the tumor microenvironment and secrete a large number of exosomes. MSCs have been found to have extensive medical value, and related research has generated an increasing number of insights. The current research shows that MSCs can mobilize peripheral blood, adipose tissue, dental pulp, and even fetal livers and lungs from bone marrow, umbilical cord blood, umbilical cords, placenta tissue cultures, and expansion in vitro. Although MSCs come from various sources, they have some common characteristics: they take the form of fibroblasts under a microscope, often being fusiform or spindle-shaped, with abundant cytoplasm, generally adherent growth characteristics, and a high proliferation rate. The surface expression markers are CD90, CD105, CD44, CD73, CD9, and very low levels of CD80, the surface markers for hematopoietic cells include CD34, CD45, CD11b, CD11c, CD14, CD19, CD79a, CD86, and the major histocompatibility complex, and the expressions of class II MHCs are all negative. MSCs secrete transforming growth factor-β, (TGF-β), hepatocyte growth factor (HGF), nitric oxide/idoxifene (NO/IDO), interleukin-10 (IL-10), IL-6, interleukin-1 receptor (IL-1Rα), human leucocyte antigen-G (HLA-G), prostaglandin E2 (PGE-2), and other soluble factors to achieve immune regulation and support hematopoiesis [3,4]. Because MSCs have the dual characteristics of directional migration to cancer sites and many foreign gene expressions, it is expected that MSCs are used to carry apoptotic genes to target tumor cells. The targeted delivery of anti-cancer drugs in mice greatly reduces the damage the drugs cause to non-tumor tissues.

In general, exosomes consist of a class of membrane-bound vesicles with diameters ranging from 50 to 150 nm from the endosomes; they also contain various proteins that are naturally taken up by human recipient cells [3,4]. Until now, compared to the direct application of stem cells to patients with different illnesses, UC-MSCs have shown several clinically advantageous characteristics, including ease of isolation, the expansion of desirable paracrine abilities, and low immunogenicity. They clearly represent an effective treatment option that does not involve invasive harvest procedures or the ethical consideration of clinical practices [5,6,7].

Furthermore, anti-inflammatory, anti-fibrosis, angiogenesis, and cardiac regeneration effects have been shown to be improved by the application of UC-MSCs, but other beneficial effects regarding clinical uses have rarely been explored.

Cancer cells lose the cycle checkpoints of normal cells, exhibit uncontrollable division and proliferation, plunder a large amount of nutrients in the body, and finally cause the body’s death. The metastasis of cancer cells is the leading reason for cancer deaths worldwide; there were expected to be 14.1 million new cancer cases globally by the early 2010s [8,9]. Hepatocellular cancers (HCCs) are the sixth most common and the second most fatal type of cancer worldwide [10,11], highlighting the urgent need to develop a novel strategy to effectively defeat different types of HCCs. Among the different types of HCCs, the hepatoblastoma type of cancer is also the most common pediatric liver malignancy; it often presents in the first years of life and exhibits markedly low responsiveness to chemotherapy [12,13,14,15]. Therefore, the current study uses the model cancer cell line HepG2, which is derived from a patient with hepatoblastoma, to explore the possible effectiveness of UC-MSC treatments in terms of impeding the perpetual growth of hepatocellular cancer model cells (HepG2 cells). Furthermore, the current study aimed to explore the molecular mechanism of genes that are involved in the death of key cancer cells; we also determined the key signaling pathways that prevent the proliferation of hepatocellular cancer cells.

## 2. Materials and Methods

### 2.1. Exosome Extraction and Examination

Mesenchymal-stem-cell (MSC)-derived exosomes (UC-MSCs) were cultured using Dulbecco’s modified Eagle’s medium (New York, NY, USA) supplemented with 10% fetal bovine serum (New York, NY, USA) in a 5% CO_2_ humidified incubator at 37 °C. Before the extraction of the MSC-derived exosomes (UC-MSCs), the UC-MSCs were washed with phosphate buffer solution (New York, NY, USA) three times. The UC-MSCs were isolated using a total exosome isolation kit (Invitrogen, Waltham, MA, USA). In brief, the cell culture was centrifuged at 2000× *g* for 30 min to harvest the UC-MSCs. Then, the supernatant was transferred into a new high-speed centrifugal tube with an extraction solution (culture media:exosome isolation reagent = 2:1); it was then mixed well by vortexing and incubated at 4 °C overnight. The above samples were centrifuged in a refrigerated high-speed centrifuge at 10,000× *g* for 1 h at 4 °C, and the supernatant was transferred to an ultracentrifuge tube and centrifuged in an ultracentrifuge at 100,000× *g* for 2 h at 4 °C. The pellet was washed by centrifuging at 100,000× *g* for 2 h at 4 °C. After centrifugation, the supernatant was discarded and the extracted exosomes were located at the bottom of the centrifugal tube (not visible in most cases). The exosomes were resuspended with PBS (25 μL PBS/1 mL culture media) and were ready for downstream analysis. If not used, the extracted exosomes were stored at −80 °C.

Transmission electron microscopy (JEOL, Tokyo, Japan) was used to observe the morphology of the exosomes. Nanoparticle tracking analysis (NTA) was used to detect the size distribution and concentration of the exosomes via ZetaView (Particle Metrix, Meerbusch, Germany). To provide a representative size distribution profile for the exosomes, videos of three replicates of the size distribution profiles were averaged. The protein concentration of the exosomes was measured using a bicinchoninic acid (BCA) protein assay kit (Beyotime, Shanghai, China). The exosome-specific expression biomarkers CD109 and TSG101 were analyzed using Western blot analysis [16,17].

### 2.2. Exosome Proteome Analysis

Nano-LC-MS/MS was used to analyze the protein components of the UC-MSCs. In brief, to perform protein digestion, 100 µg of the protein extract was adjusted to a final concentration of about 1 µg/uL with 50 mM ammonium bicarbonate (pH 8.0). The test protein samples were further desalted using the C18 tip and dried using a speed vacuum; then, each was dissolved in 20 µL water containing 0.1% formic acid for the LC-MS/MS analysis. The final samples were analyzed using a Nano-LC-MS/MS system consisting of an Easy nLC 1000 (Thermo Fisher Scientific, Waltham, MA, USA) and an Orbitrap Fusion Lumos mass spectrometer (Thermo Fisher Scientific, USA) equipped with a nano-electrospray source (Thermo Fisher Scientific, USA). During the chromatographic separation, the digested peptides in the samples were separated on an analytical C18 column (75 µm and 50 cm PepMap RSLC, Thermo Fisher Scientific) at a flow rate of 300 nL/min, and the analysis was performed using the data-dependent mode, automatically switching between MS1 and MS2. The MS data were acquired using the following parameters: MS1 spectral data (400–1600 *m*/*z*) were acquired using the Orbitrap with a maximum ion injection time of 100 ms at a resolution of 120,000 and an automatic gain control (AGC) target value of 4.0 × 10^5^. MS2 spectra were acquired using the Orbitrap mass analyzer at a resolution of 30,000 with high-energy collision dissociation (HCD) of 27% normalized collision energy and an AGC target value of 5.0 × 10^4^, with a maximum ion injection time of 54 ms. Previously fragmented ions were excluded for 30 s.

The identification of peptides was performed against the Uniprot human database with a precursor mass error of 5 ppm and a fragment ion mass error of 600 ppm, and then the genes of the target proteins were predicted based on the identified Uniprot domains. The output data files were further filtered and sorted with two or more peptide assignments for protein identification, with a false positive rate of less than 0.01 using DTASelect (Scripps Research Institute, San Diego, CA, USA).

### 2.3. Cell Culture

The HepG2 cell line was used as a liver cancer cell model in the experiments. The HepG2 culture is a hepatoblastoma-derived cell line and was obtained from the collection of ATCC (Manassas, VA, USA). DMEM/F12 supplemented with 10% fetal bovine serum (FBS) was used to culture the HepG2 cell with the addition of 100 units/mL penicillin/streptomycin (Gibco, Carlsbad, CA, USA). Cultural conditions were maintained in a humidified CO_2_ incubator under standard conditions (5% CO_2_, 37 °C). Before examining the cytotoxicity of UC-MSCs and DOX, the HepG2 cells were simultaneously treated with different concentrations of UC-MSCs at 10, 100, and 1000 µg protein/mL and 25 μM DOX in a 1% O_2_ incubator for 48 h, according to the group.

### 2.4. Cytotoxicity Assay

A Cell Counting Kit-8 (CCK-8) reagent (Dojindo China Co., Ltd., Shanghai, China) was used, following the manufacturer’s instructions. HepG2 cells from the different treatment groups were evenly distributed on a 96-well plate, with three replicate samples per group. The CCK-8 reagent was added 24 h after the cell treatment. After appropriate incubation, the absorbance was measured at 450 nm using a multi-function microplate reader. The above preconditioned HepG2 cells were harvested using 0.25% trypsinization (without ethylenediaminetetraacetic acid) and transferred to Eppendorf tubes. They were washed twice with PBS. After the HepG2 cells were resuspended with 300 μL binding buffer of annexin V and PI, 5 μL annexin V and 5 μL PI were added. After incubation for 10 min at room temperature in the dark, the results were examined using a flow cytometer (BD Accuri C6, BD Biosciences, Piscataway, NJ, USA).

### 2.5. RNA-Seq Data Analysis

The HepG2 cells were collected and immediately frozen in liquid nitrogen before being stored at −80 °C. Total RNA was extracted using an RNeasy Plus Mini Kit (Qiagen, Hilden, Germany). RNA purity was checked using a NanoPhotometer^®^ spectrophotometer (IMPLEN, Westlake Village, CA, USA). RNA concentration was determined using a Qubit^®^ RNA Assay Kit and a Qubit^®^ 2.0 Fluorometer (Life Technologies, Carlsbad, CA, USA). RNA integrity was assessed using the RNA Nano 6000 Assay Kit of the Agilent Bioanalyzer 2100 system (Agilent Technologies, Santa Clara, CA, USA). A total of 3 μg RNA per sample was used to prepare the RNA library. The NEBNext Poly (A) mRNA Magnetic Isolation Module (New England BioLabs, Ipswich, MA, USA) was used to isolate the mRNA. Subsequently, cDNA was synthesized using a TruSeq RNA Library Prep Kit v2 (Illumina, San Diego, CA, USA) before cDNA fragmentation and fragment purification. The sequencing libraries were constructed with the purified fragmented cDNA using a NEBNext^®^ Ultra™ RNA Library Prep Kit for Illumina (New England BioLabs, MA, USA), following the manufacturer’s recommendations. The prepared libraries were sequenced on an Illumina NovaSeq 6000 platform (Illumina, USA), and about 6 Gb of sequencing data was acquired per sample. Paired-end raw reads of RNA-seq data were cleaned using Trimmomatic v0.39. Gene expression levels were determined via HISAT2 v2.0.5 and StringTie2 v2.0.4. Differential expression analysis was performed using DESeq v1.10.1. DESeq provides statistical routines for determining significantly differentially expressed genes (DEGs) using an adjusted *p*-value < 0.05 based on Benjamini and Hochberg’s approach. Enrichment analysis of the DEGs was performed against the GO (Gene Ontology) database and the KEGG (Kyoto Encyclopedia of Genes and Genomes) database. For the WGCNA analysis (weighted gene co-expression network analysis), highly co-expressed gene modules were inferred from the FPKM values (FPKM > 10) using the R package WGCNA. The WGCNA network was constructed using a minimal module size of 100, an unsigned topological overlap matrix type, a soft power of 12, and a merge cut height of 0.45.

### 2.6. Identification and Analysis of Metabolites via LC-MS

The HepG2 cell samples were transferred into a 2 mL EP tube; 1 mL of an acetonitrile:methanol:H_2_O mixed solution (2:2:1, *v*/*v*/*v*) was carefully added and the mixture was vortexed for 30 s. The tubes were placed into liquid nitrogen for 5 min and thawed at room temperature; then, they were placed in the tissue grinder for 2 min at 60 Hz. The above operation was repeated twice. Centrifugation was conducted at 4 °C for 10 min at 12,000 rpm, and then 850 μL of the supernatant from each sample was transferred into another 2 mL centrifuge tube. The samples were concentrated to dry in a vacuum; the samples were then dissolved with 300 μL of 2-chlorobenzalanine solution (4 ppm) prepared with acetonitrile: 0.1% FA (1:9 *v*/*v*) (−20 °C), and the supernatant was filtered through a 0.22 μm membrane to obtain the prepared samples for LC-MS. Finally, 20 μL from each sample was taken for use in quality control (QC) samples.

For the metabolite analysis, full-scan MS spectra at a resolution of 100,000 (defined as *m*/*z* 400) were collected on a Thermo Scientific LTQ-Orbitrap Velos mass spectrometer in both the positive and negative ionization modes. Scans were collected from *m*/*z* 200 to *m*/*z* 2000. For each analysis, 5 μL of the sample was directly introduced via flow injection (no LC column) at 1 L/min using a HESI ion source equipped with a low-flow ESI needle. The sample and injection solvent were 2:1 (*v*:*v*) isopropanol:methanol containing 20 mmol/L ammonium formate. The conditions were set as follows: the spray voltage was 4.0 kV, the ion transfer tube temperature was 200 °C, the S-lens value was 50%, and the ion trap fill time was 100 ms. Following the MS data acquisition, mass recalibration was performed using Thermo Xcalibur software v.4.2 according to the manufacturer’s instructions, using the theoretical computed masses. MS/MS confirmation of the ions of interest was obtained using higher-energy collisional dissociation (HCD) MS/MS at a resolution of 100,000 and a normalized collision energy of 25 for the positive ion mode and 60 for the negative ion mode. The metabolites were identified using the Mass Spectrum Analysis (MSA) v.1.0 software’s linear fit algorithm, in conjunction with an in-house database of hypothetical human metabolites, for automated peak detection. The relative quantification of abundance between samples was performed according to the peak area of each identified compound.

### 2.7. Statistical Analysis

Statistical analysis was conducted using one-way ANOVA (Dunnett’s multiple comparisons test) using Sigma Plot 11.0, USA. The level of statistical significance chosen was *p* < 0.05, unless otherwise stated.

## 3. Results

### 3.1. The Morphology, Representative Size Distribution, and Protein Component Analysis of UC-MSCs

**Figure 1:**
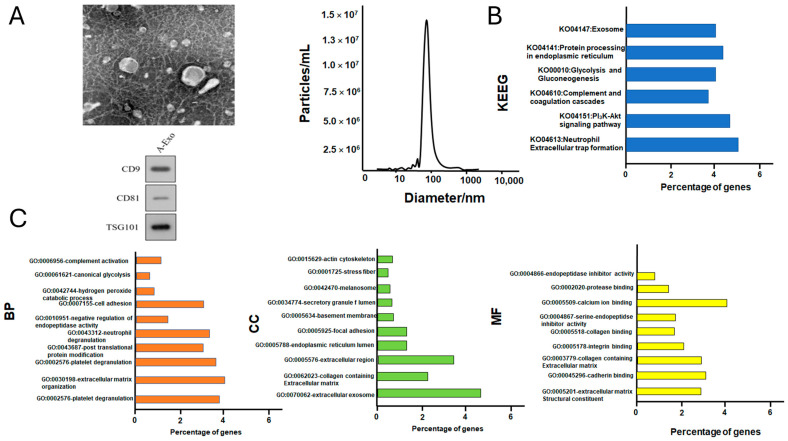
The morphology, representative size distribution, and protein component analysis of the UC-MSCs. (**A**) Examination of UC-MSCs-EXO morphology and size distribution; (**B**) KEGG test of UC-MSCs-EXO protein components; (**C**) UC-MSCs-EXO protein component GO test (biological process in GO category/cellular component in GO category/molecular function in GO category).

First, the UC-MSCs were examined with TEM and observed to have the typical spheroid morphology of exosomes (Figure 1). Second, the representative size distribution of the exosomes was verified at the mean diameter of around 134 nm using the NTA assay (Figure 1). The protein concentrations of the prepared exosomes were carefully adjusted to 10 µg/mL, 100 µg/mL, and 1000 µg/mL for the treatments of the HepG2 cell cultures.

The 290 protein components of the UC-MSCs were identified with more than five hits and further investigated using KEGG (Kyoto Encyclopedia of Genes and Genomes) pathway enrichment analysis and GO (Gene Ontology) functional enrichment analysis, annotating the key pathways in KEGG and the biological functions in GO (Figure 1B,C). According to Figure 1B, the exosomal proteins (adjusted *p*-value < 0.05) were significantly enriched in different KEGG pathways, including the KEGG terms, tight junction (ko04530, 2.4%), focal adhesion (ko04510, 2%), cardiac muscle contraction (ko04260, 1.6%), the PI3K-Akt signaling pathway (ko04151, 3.5%), and complement and coagulation cascades (ko04610, 4.3%). Complements, such as C3 and C5, were detected in the samples. For the GO enrichment analysis (Figure 1C) conducted in GO_BP (the biological process in the GO category), the significant key terms included cell-matrix-related terms and neutrophil degradation (GO:0043312, 11.8%); in GO_CC (the cellular component in the GO category), the cellular structure was mainly anchored with the GO_CC term, extracellular exosomes (GO0070062, 58%), and the other key cellular structures, including the regulation of the actin cytoskeleton (GO0015629, 5.2% and GO0001725, 4.8%), cell adhesion components (GO:0005788, 14.1%), and the regulation of the extracellular matrix (GO0031012, 9.3%), all of which are known to regulate the formation and transportation of exosomes; in GO-MF (the molecular function in the GO category), the GO_MF terms were mainly enriched by the key terms, including several types of binding activities by Ca^2+^ and actin, extracellular matrix components (GO0005178, 5.5%, etc.), and serine-type endopeptidase inhibitor activity (GO:0004867, 4,7%). The enriched terms indicated that the exosomal protein components have the potential to activate complementary pathways, inhibit endopeptidase, and regulate the extracellular matrix.

### 3.2. Cytotoxicity Test of the UC-MSCs Treatment of HepG2 Cells

**Figure 2:**
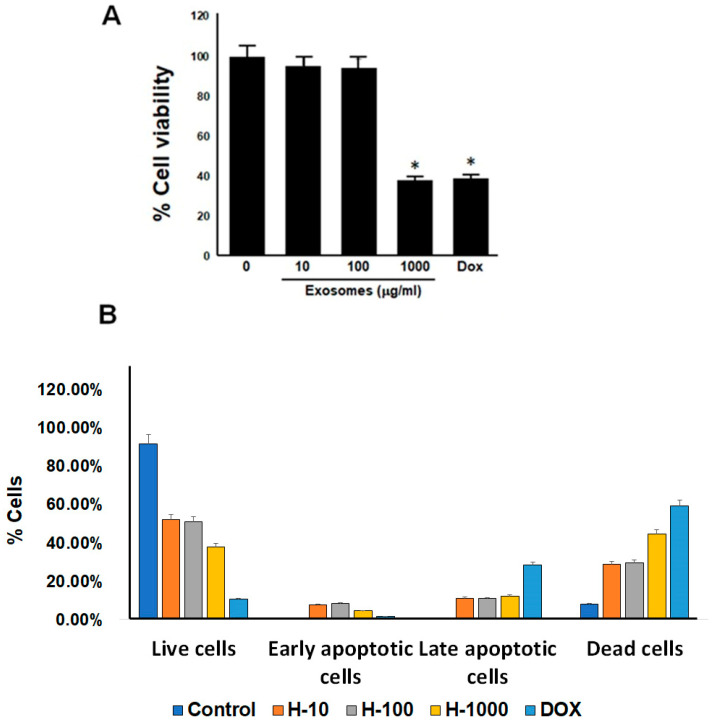
Cytotoxicity test of UC-MSC treatments of HepG2 cells. (**A**) In the examination of cytotoxicity, HepG2 cell cultures were divided into five groups, and examined with CCK8 assays. Substantial reduction of 65% and 61% in HepG2 survival was observed with both treatments of DOX(doxorubicin) and 1000 µg/mL UC-MSCs after 24 h of exposure. (**B**) The flow cytometry analysis of HepG2 cells. * *p* < 0.05.

In the examination of cytotoxicity, the HepG2 cell cultures were divided into five groups: control (control, untreated HepG2 cells), H_10 (HepG2 cells treated with 10 µg/mL UC-MSCs), H_100 (HepG2 cells treated with 100 µg/mL UC-MSCs), H_1000 (HepG2 cells treated with 1000 µg/mL UC-MSCs), and positive (100 µg/mL DOX treated HepG2 cells) according to CCK8 assays. Substantial reductions of 65% and 61% in HepG2 survival were observed under the DOX and 1000 µg/mL UC-MSC treatments, respectively, after 24 h of exposure (Figure 2A). Furthermore, the flow cytometry analysis of the treated HepG2 cells indicated that the cell death caused by the UC-MSC treatments was most likely due to direct cell degradation, as the treatments with low concentrations of UC-MSCs led to a constant percentage of both early and late apoptotic cells compared to the high concentration of UC-MSCs (1000 µg/mL). However, unlike DOX, they could trigger the process of cell apoptosis, as demonstrated by the higher percentage of late apoptotic cells, 28.49%, compared to that resulting from the UC-MSC treatments (Figure 2B).

### 3.3. Analysis of Transcription Profiles in the Five Groups of HepG2 Cells

**Figure 3:**
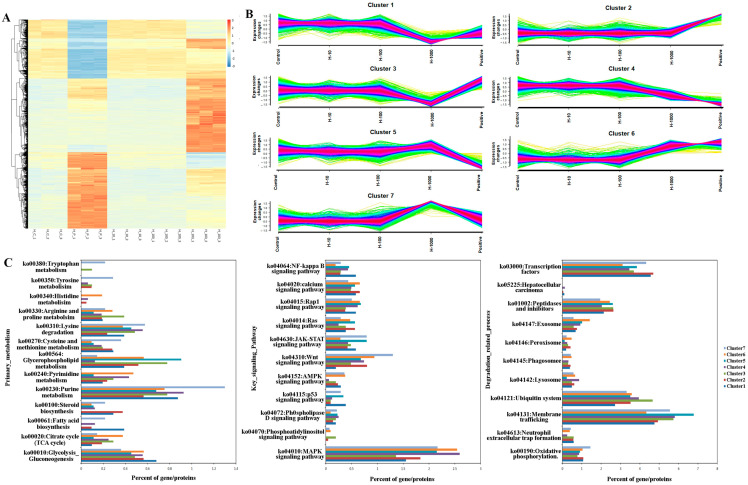
Analysis of transcription profiles in five groups of HepG2 cells. (**A**) A total of 53,224 transcripts were identified via deep transcriptome sequencing; (**B**) all the expressed genes of the HepG2 cells were subjected to one-way ANOVA analysis to identify the differentially expressed genes (ANOVA-DEGs); (**C**) KEGG pathway enrichment analysis.

A total of 19,967 transcripts were identified based on deep transcriptome sequencing, and 12,743 transcripts were expressed with more than 50 reads in at least one of the samples. Among the five groups, all the expressed genes of the HepG2 cells were subjected to one-way ANOVA analysis to identify the differentially expressed genes (ANOVA-DEGs). The hierarchical clustering of these genes is demonstrated in Figure 3A,B. Both the H_P group and the H_1000 group exhibit significantly different expression patterns compared to the other three groups, but there are still notable differences between the H_P and H_1000 groups, indicating that diverse mechanisms inhibit the growth of HepG2 cells (see Supplemental Figure S3). Additionally, the expression patterns among the five groups enabled us to accurately classify seven distinct expression profiles (Figure 3B). Regarding changes in expression levels, Cluster 3 represents the specific gene sets only for the downregulated genes in the H-1000 group, and Cluster 7 represents the specific gene sets only for upregulated genes in the H-1000 group, (Figure 3B). Subsequently, KEGG pathway enrichment analysis was used for all seven clusters for three major categories: primary metabolism, key signaling pathways, and degradation-related pathways. Cluster 3 was more enriched in the ubiquitin system (ko04121, 4.66%); see Figure 3C. Cluster 7 was more enriched in the purine metabolism (ko00230, 1.298%), in the primary metabolism and Wnt signaling pathway (ko04310, 1.298%), and in the degradation-related pathways (Figure 3C). All in all, there is no specific trend in the global analysis of transcriptomes that reflects the inhibition of HepG2 cell growth.

### 3.4. Transcriptome Analysis Identified Several Key Hub Genes Using WGCNA

**Figure 4:**
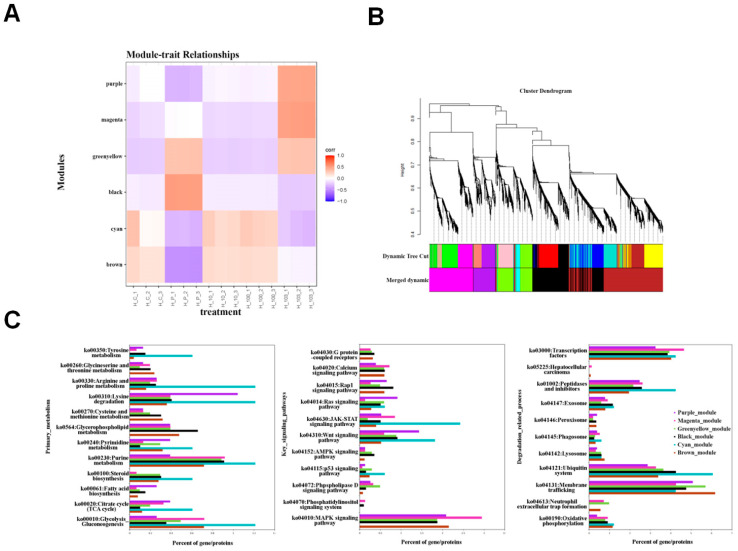
Transcriptome analysis using the WGCNA protocol. (**A**,**B**) ANOVA-DEGs from the five groups were further subjected to WGCNA analysis and divided into six color modules with distinct expression profiles; (**C**) the key hub upregulated and downregulated genes were further identified through the analysis of the protein–protein interaction network, and then the genes were subjected to KEGG pathway enrichment analysis.

ANOVA-DEGs from the five groups were further subjected to WGCNA analysis (weighted gene co-expression network analysis) and divided into six color modules with distinct expression profiles (Figure 4A,B). KEGG pathway enrichment analysis was applied to the genes in each color module and the key KEGG pathways were divided into three different categories, including the primary metabolism, key signaling pathways, and degradation-related pathways (Figure 4C). In the primary metabolism category, the genes from the cyan and brown modules were downregulated for both the positive and H_1000 groups and were more enriched in the purine metabolism and several amino acid metabolisms (Figure 4A,C). Regarding the key signaling pathways, the downregulated genes in cyan for both the positive and H_1000 groups were also enriched for the JAK-STAT signaling pathways (ko04630, 2.6%) and Wnt signaling pathway (ko04310, 1.8%); the upregulated genes in the purple module and magenta module, for H_1000 only, were enriched in the Ras and MAPK-related signaling pathways, transcription factors (ko03000, 4.7%), and the MAPK signaling pathway (ko04010, 2.9%) (Figure 4C). As for degradation-related processes, the downregulated genes in the cyan module for both the positive group and the H_1000 group were similarly enriched in the Ubiquitin system (ko04121, 3.3%). Key hub genes were further identified as having the most protein–protein interactions via protein interaction network analysis (Supplemental Figures S4 and S5). The upregulated hub genes were identified with five genes in the magenta module, such as FOSL1 and BTK, which play a role in signaling transduction, and with only one gene in the purple module, which plays a role in solute transportation. The main downregulated hub genes were two genes and four genes for the brown module and the cyan module, respectively, and their gene annotations suggest that they are involved in the regulation of tight junctions, TEX14, and the microtubule cytoskeleton, TUBA1B.

### 3.5. Key Pathways Identified Through the Enrichment Analysis of Key DEGs Between Groups

Compared to the Control group, differentially expressed genes (DEGs) in the H_1000 group and positive group were identified based on the criteria of fold change (FC) > 2; with a *p*-value < 0.05. A total of 1138 DEGs were identified, with only one downregulated gene and 1137 upregulated genes for the H_1000 group. In contrast, the 1600 DEGs were extracted in search of greener pastures for their livestock.

**Figure 5:**
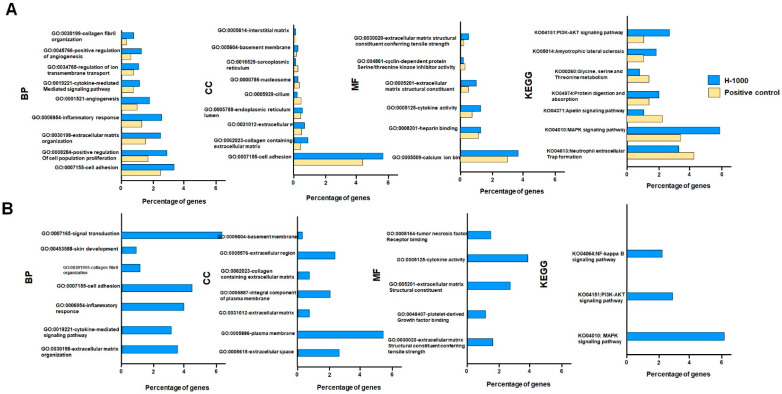
DEGs in the H_1000 group (**A**) and positive control group (**B**) compared to the control group were further subjected to the GO functional enrichment analysis.

A comparison between the positive group and the control group, with 609 downregulated genes and 991 upregulated genes (therefore, the upregulated DEGs from both comparisons were further subjected to the KEGG pathway enrichment analysis and GO functional enrichment analysis). In terms of GO_BP, the key terms for the DEGs were mainly enriched for the comparison between the H_1000 group and the control group, including blood tissue development, the regulation of the extracellular matrix, and cell proliferation (Figure 5A). The DEGs were only significantly enriched for the comparison between the H_1000 group and the control group in several GO_CC terms, including the regulation of extracellular matrix structures and different cellular membrane organelles (Figure 5A). In GO-MF, the key terms were enriched for the regulation of the extracellular matrix, signaling molecule binding activities, and cytokine activities in the comparison between the H_1000 group and the control group; however, they were enriched for cyclin-dependent serine threonine inhibitor activity in the comparison between the positive group and the control group (Figure 5A). In the KEGG pathway enrichment analysis (Figure 5A), one key pathway was identified to be significantly enriched with both comparisons (ko04613: neutrophil extracellular trap formation). Furthermore, the analysis of the pathway ko04613 (Supplemental Figure S6) showed that the upregulated genes in the S_1000 group were different from those in the positive group, even though both treatments demonstrated the involvement of histone-mediated cellular processes and the activation of nicotinamide adenine dinucleotide phosphate (NADPH)-oxidase. On the other hand, the S_1000 group uniquely exhibited signaling transduction via the triggering of the upregulation of the iC3b, C3b, and C3b receptors (Supplemental Figure S6). This refers to the recognized processes triggered by microorganisms and endogenous stimuli, such as damage-associated molecular patterns and immune complexes. Additionally, the analysis of “complement and coagulation cascades” and “Amoebiasis” in KEGG showed that the protein components from the UC-MSCs were combined with the upregulated genes in the S_1000 group to activate these two canonical pathways (Supplemental Figures S7 and S8).

### 3.6. Metabolite Profiles in Different Groups of HepG2 Cells

**Figure 6:**
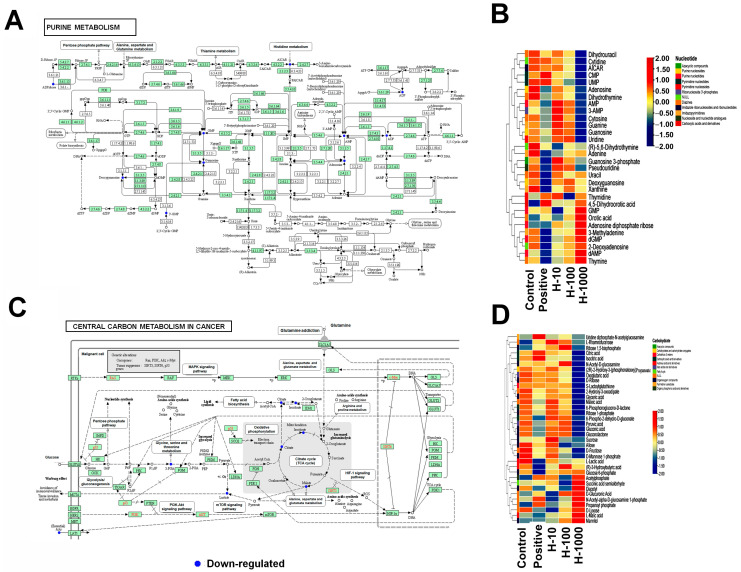
Metabolite profiles in different groups of HepG2 cells. (**A**,**B**) Upregulated metabolites are enriched in the pathways of purine metabolism and transport-related compounds; (**C**,**D**) downregulated metabolites are more specifically enriched in the pathways of hsa00330 Arg, Pro degradation, etc.

**Figure 7:**
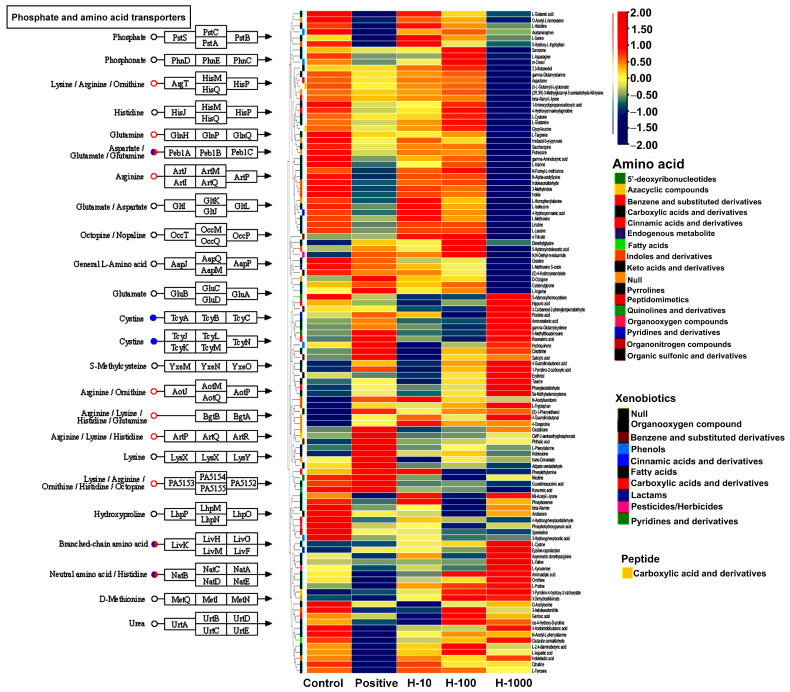
The metabolisms of key amino acids.

In total, 286 metabolites from the HepG2 cells under different treatments were identified based on MS1 and MS2 information, as described in the methods section; differential quantified metabolites were determined according to the criteria of an adjusted *p*-value < 0.05 and fold change >1.1 for upregulation or <0.9 for downregulation. The identified metabolites could mostly be classified as amino acids, lipids, nucleotides, carbohydrates, and peptides. In comparison to the control group, in the H_1000 group, the downregulated metabolites were mainly enriched in the KEGG pathways, including “purine metabolism” and the TCA cycle of “central carbon metabolism in cancer” (Figure 6); in contrast, several metabolisms of key amino acids, except for cystine, were more upregulated (Figure 7), which is probably related to the degradation of proteins in the H_1000 group of HepG2 cells.

## 4. Discussion

In this study, we observed that high concentrations of UC-MSC-derived exosomes significantly reduced HepG2 cell viability by 65%. Proteomic and transcriptomic analyses revealed the activation of pathways associated with neutrophil extracellular trap (NET) formation in HepG2 cells. NETs, composed of decondensed chromatin and granular proteins, are traditionally recognized as a neutrophil defense mechanism against pathogens. Recent studies have demonstrated that NETs also play a role in tumor biology, contributing to cancer progression, metastasis, and resistance to therapies. Additionally, NETs can influence cancer progression through immunosuppression and facilitation of metastasis. Therefore, we hypothesize that UC-MSC-derived exosomes may induce mechanisms analogous to NET formation, leading to the degradation and death of HepG2 cells. Further experimental validation is required to confirm whether the cell death induced by UC-MSC-derived exosomes in HepG2 cells is related to NETosis.

**Figure 8:**
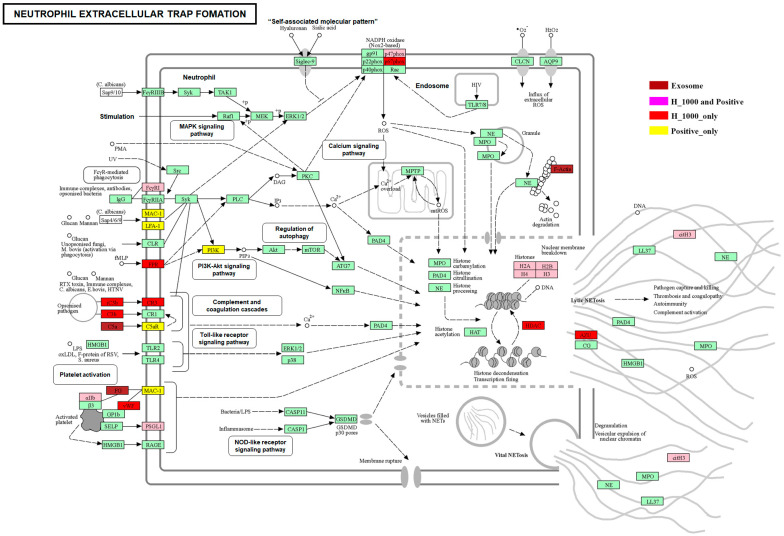
Transcriptome of UC-MSC-treated HepG2 cells.

The analysis of the protein components in the UC-MSCs was combined with a metabolite analysis and a transcriptome analysis of the HepG2 cells under different treatments (Figure 8), with the results clearly suggesting that the action of UC-MSCs mimicked one of the mechanisms of neutrophil action [18,19]: the formation of neutrophil extracellular traps (NETs). This stimulated the formation of the extracellular complex and intracellular nucleolus structures composed of chromatin coated with histones, triggering direct cell death in the HepG2 cells treated with high concentrations of UC-MSCs. The evidence from the transcriptomes of these treated HepG2 cells indicates that several of the key signaling processing involved in mediating the cellular degradation cues by UC-MSCs were involved in the PI3K-Akt signaling pathway [20,21,22,23], complemental coagulation cascades, and the Toll-like receptor signaling pathway (Figure 8). The pathways of protein digestion and absorption were shown to be commonly triggered metabolic processes in the groups treated with the positive anti-cancer drug (DOX) and UC-MSCs [24,25]. In the other aspects of the primary metabolism, the protein components of the UC-MSCs were also profoundly involved in the glycerophospholipid metabolism of the cell membrane and the downregulation of key metabolites in the central carbon metabolism in cancer.

In conclusion, our combined analyses revealed that a high concentration of UC-MSCs was able to suppress the growth of hepatocellular cancer cells [26,27] and HepG2 cells. The molecular mechanisms of its action mimicked the formation of neutrophil extracellular traps and, in turn, triggered the same mechanism of cellular destruction via canonical signaling pathways [28,29,30,31,32], such as the PI3K-Akt signaling pathway and complemental coagulation cascades. This study provided important information regarding exosomes derived from mesenchymal stem cells that will be used to explore their effect on cancer in the future.

## Data Availability

The data presented in this study are available on request from the corresponding authors.

## Supplementary Materials

The following supporting information can be downloaded at: https://www.mdpi.com/article/10.3390/cimb46120793/s1.
