# Supplementary figures and images for: Multiomics Analysis of Molecules Associated with Cancer in Mesenchymal-Stem-Cell-(MSC)-Derived Exosome-Treated Hepatocellular Carcinoma Cells
Source: Curr Issues Mol Biol. 2024 Nov 21;46(12):13296–310. doi: 10.3390/cimb46120793 (PMC11726723; doi:10.3390/cimb46120793)

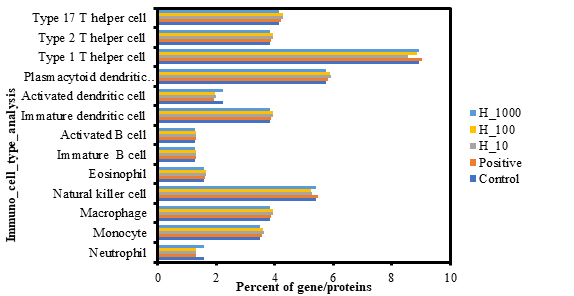

Supplement: Supplementary file 1 [file cimb-46-00793-s001.zip › suplement fig2.jpg]

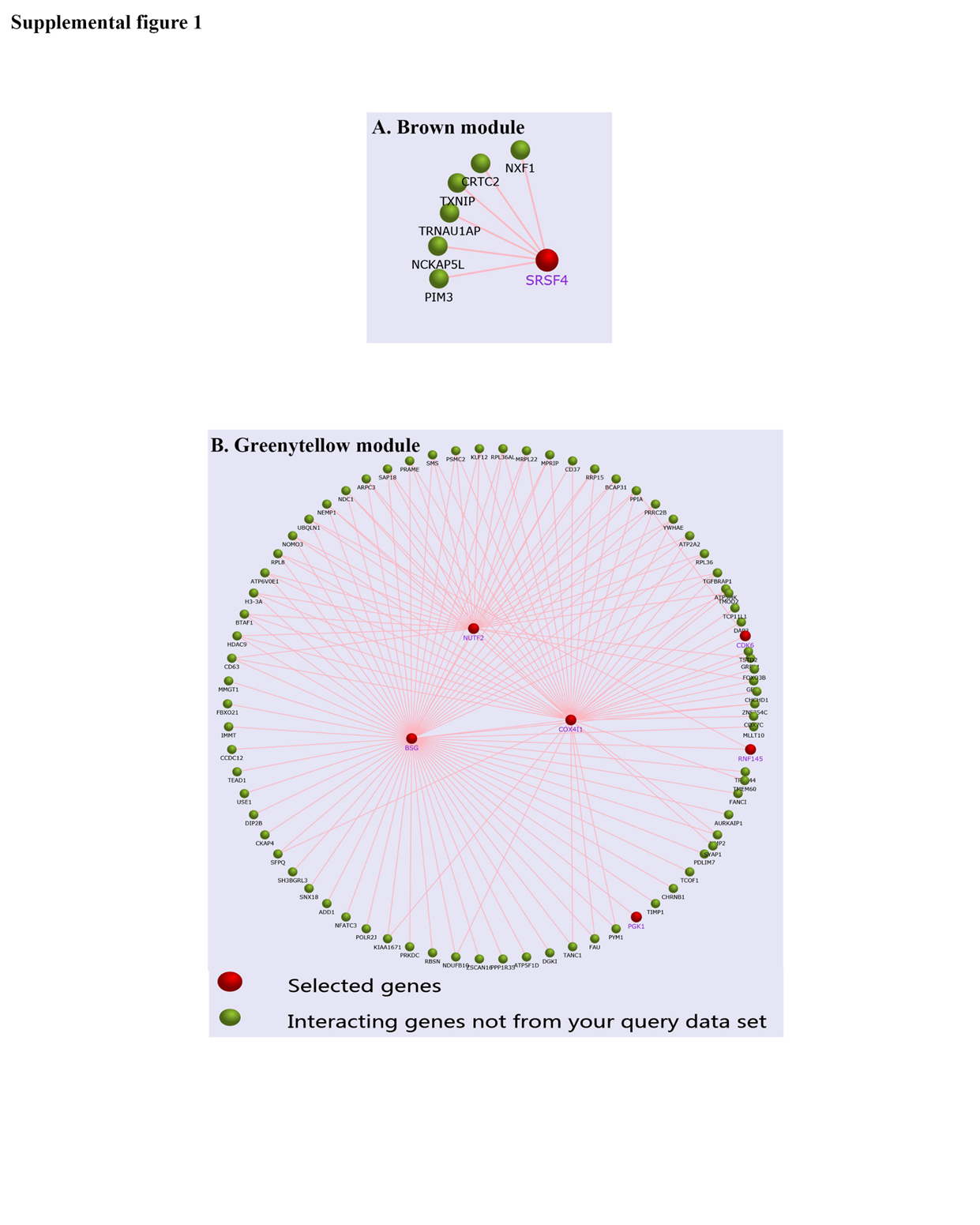

Supplement: Supplementary file 1 [file cimb-46-00793-s001.zip › suplement fig 1.jpg]

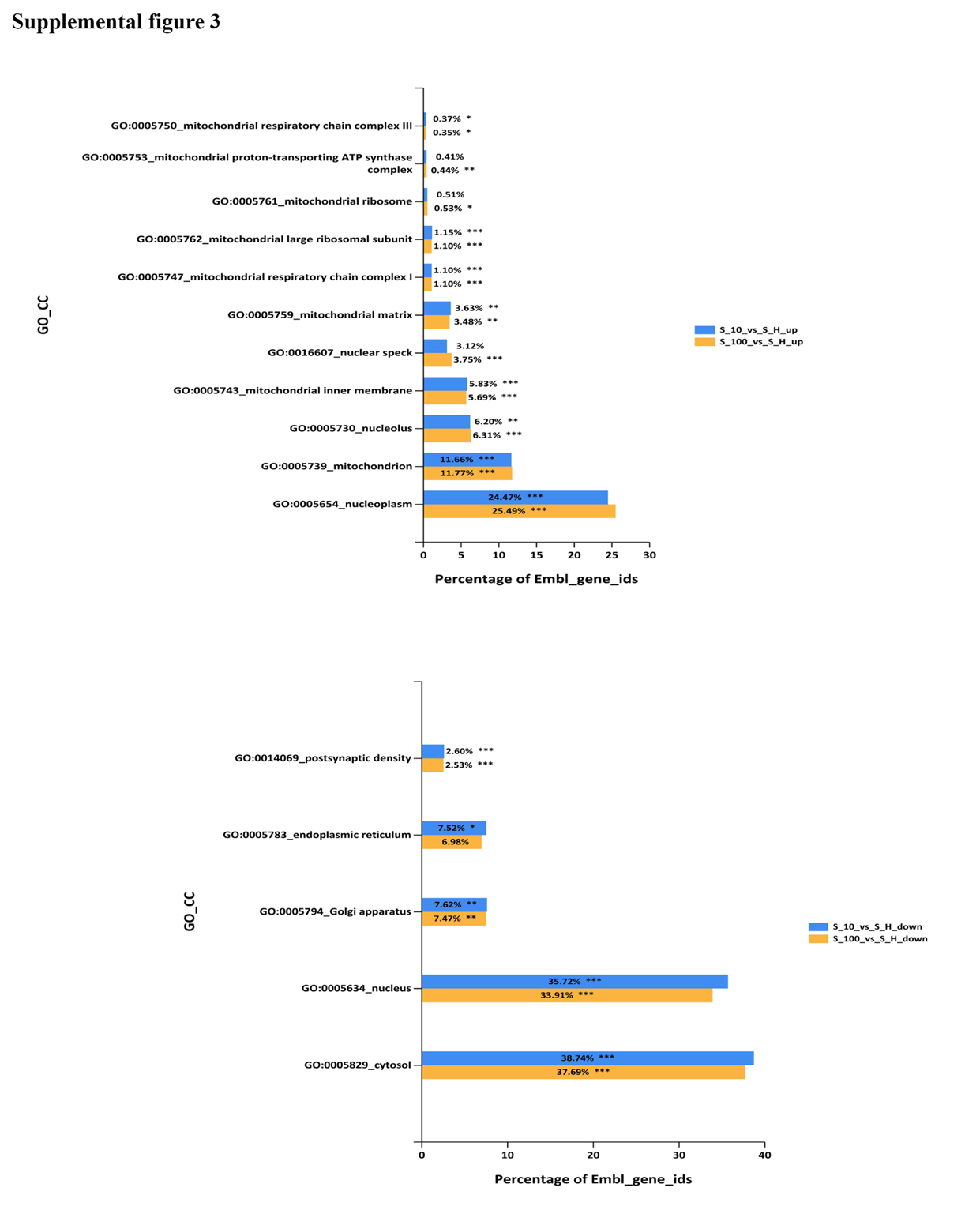

Supplement: Supplementary file 1 [file cimb-46-00793-s001.zip › suplement fig 3.jpg]

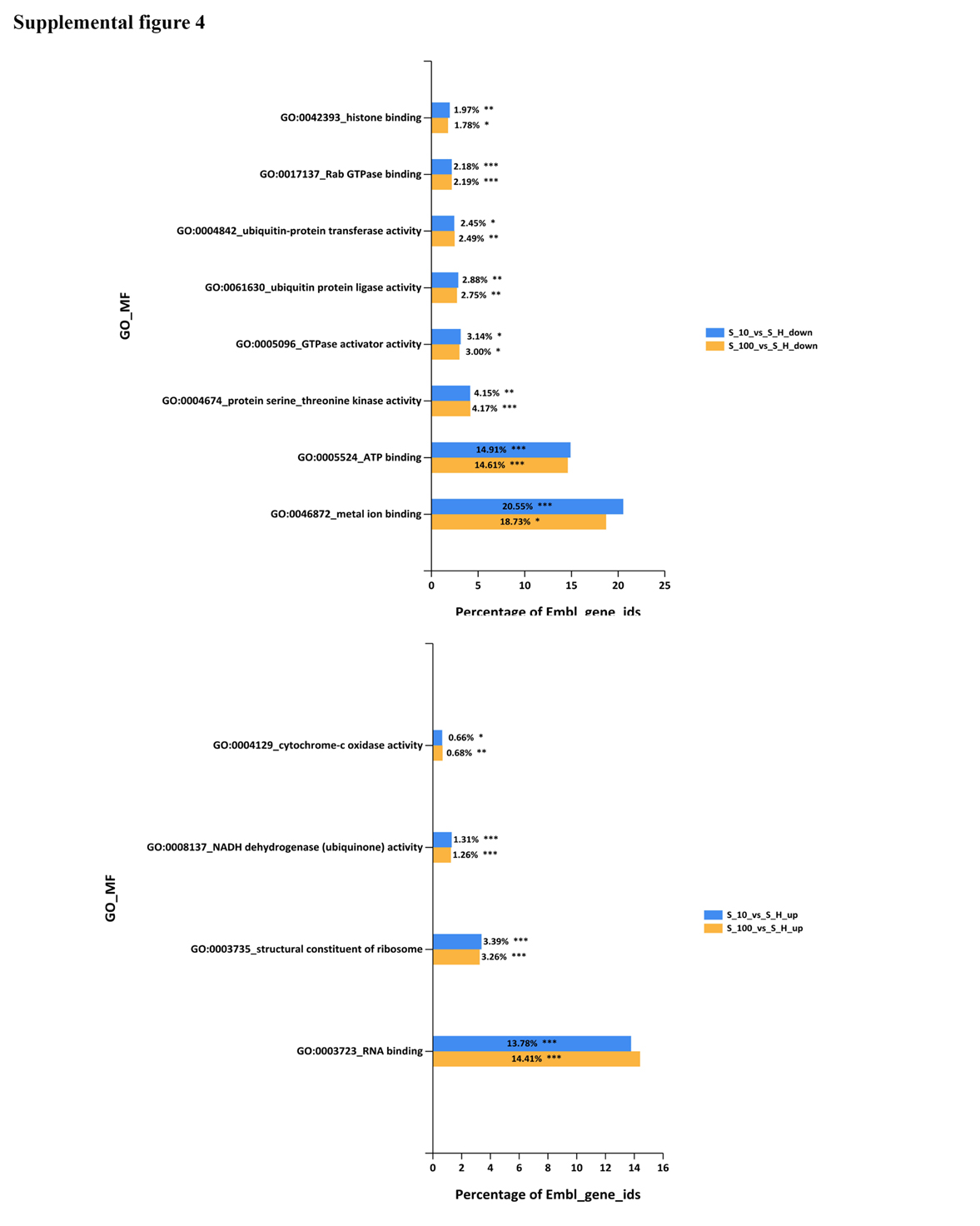

Supplement: Supplementary file 1 [file cimb-46-00793-s001.zip › suplement fig 4.jpg]

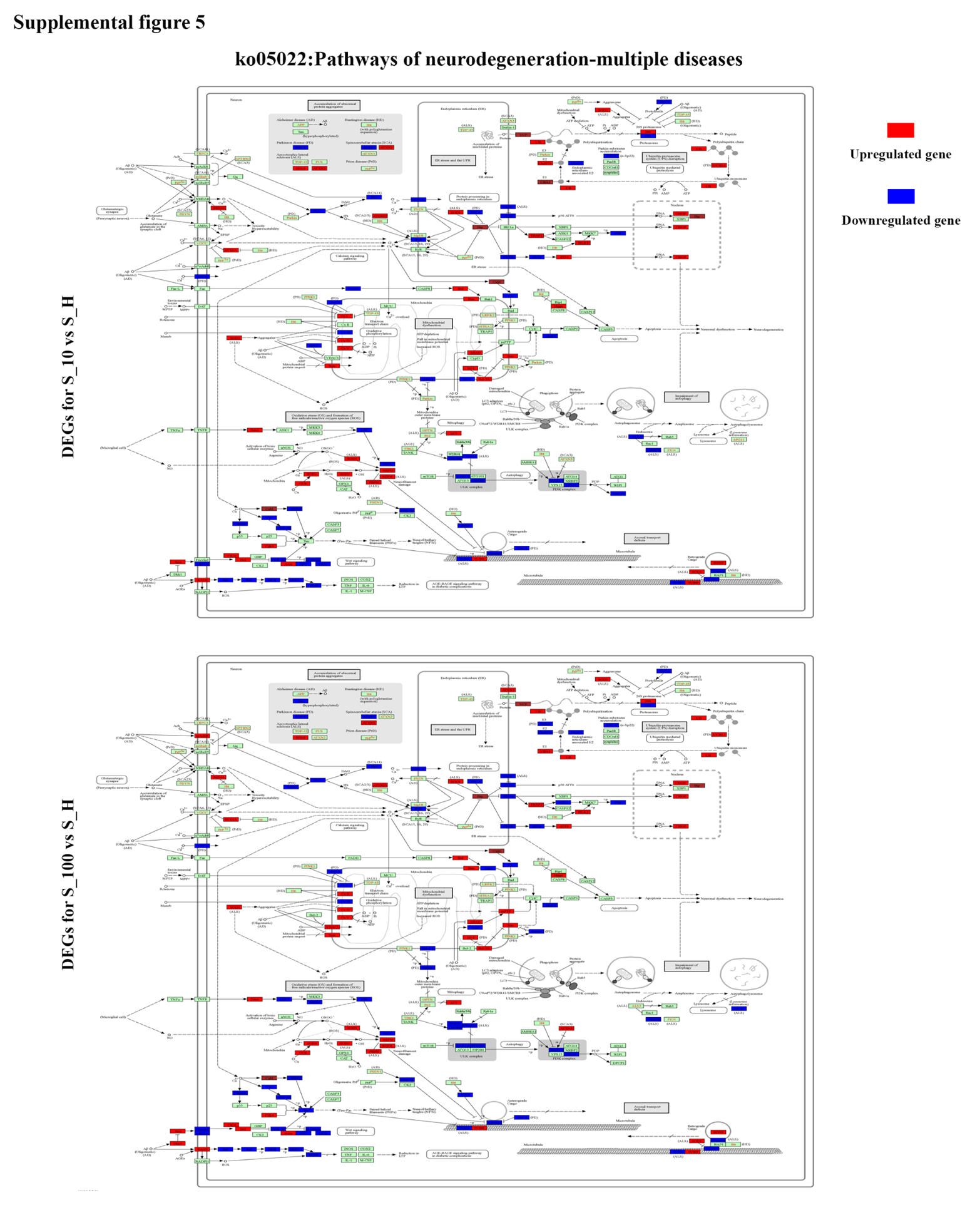

Supplement: Supplementary file 1 [file cimb-46-00793-s001.zip › suplement fig 5.jpg]

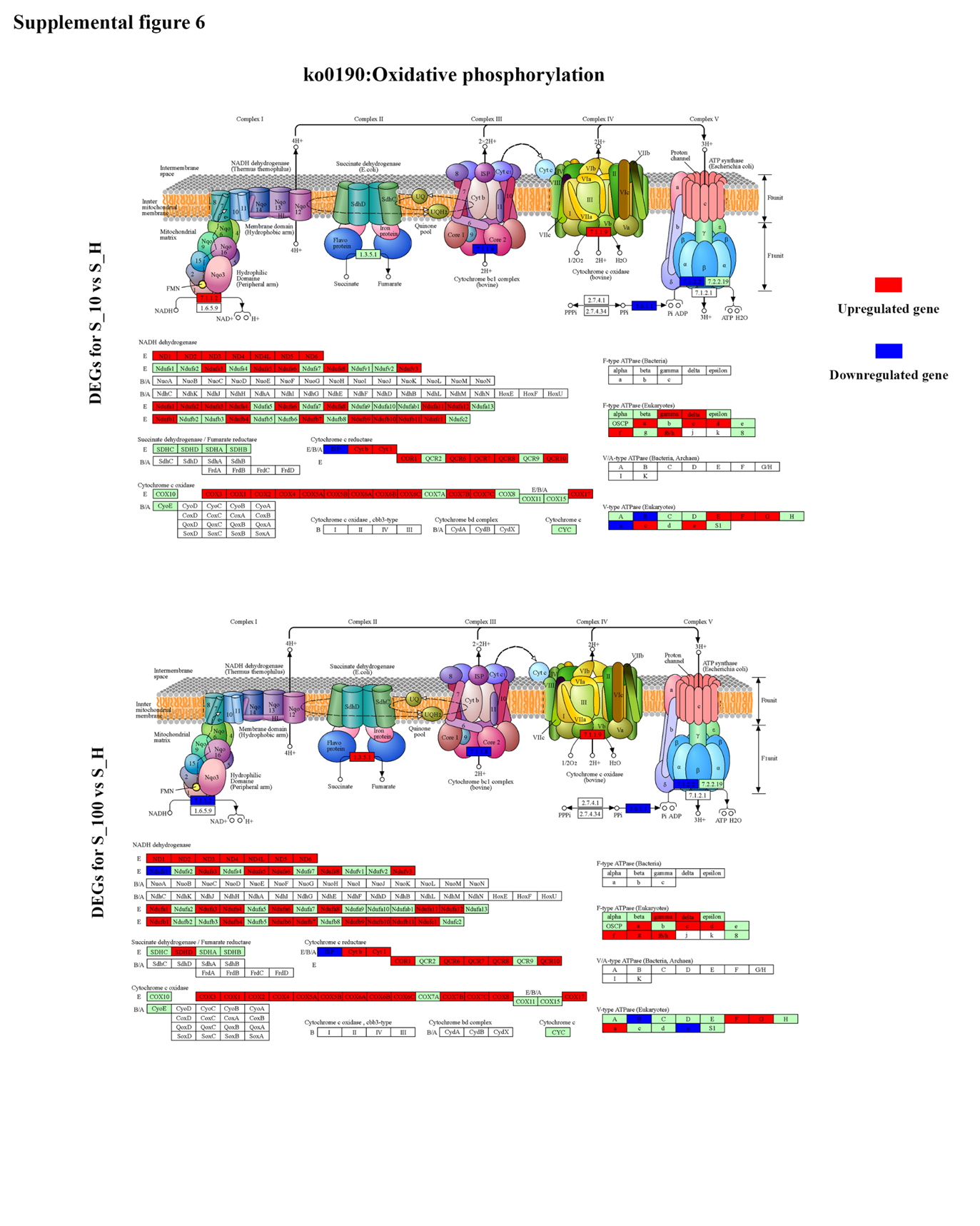

Supplement: Supplementary file 1 [file cimb-46-00793-s001.zip › suplement fig 6.jpg]

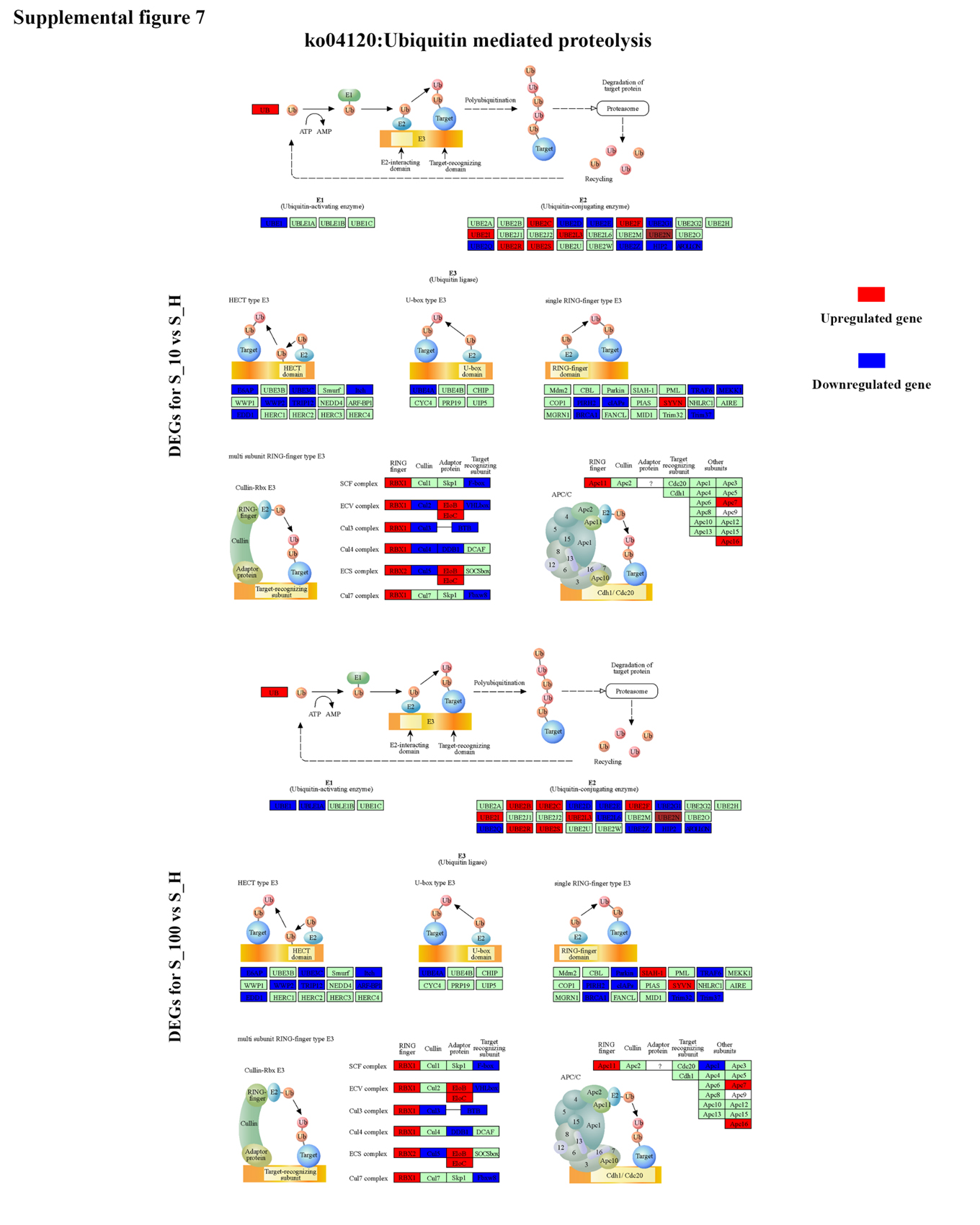

Supplement: Supplementary file 1 [file cimb-46-00793-s001.zip › suplement fig 7.jpg]
